# Car T Cells in Solid Tumors: Overcoming Obstacles

**DOI:** 10.3390/ijms25084170

**Published:** 2024-04-10

**Authors:** Joselyn Rojas-Quintero, María P. Díaz, Jim Palmar, Nataly J. Galan-Freyle, Valery Morillo, Daniel Escalona, Henry J. González-Torres, Wheeler Torres, Elkin Navarro-Quiroz, Diego Rivera-Porras, Valmore Bermúdez

**Affiliations:** 1 Medicine, Pulmonary, Critical Care, and Sleep Medicine Department, Baylor College of Medicine, Houston, TX 77030, USA; joselyn.rojasquintero@bcm.edu; 2 Facultad de Medicina, Centro de Investigaciones Endocrino—Metabólicas, Universidad del Zulia, Maracaibo 4001, Venezuelajim.palmarb@gmail.com (J.P.); valerycmb@hotmail.com (V.M.); danielescalona0397@gmail.com (D.E.); wheelertorres16@gmail.com (W.T.); 3 Centro de Investigaciones en Ciencias de la Vida, Universidad Simón Bolívar, Barranquilla 080002, Colombia; nataly.galan@unisimon.edu.co (N.J.G.-F.); elkin.navarro@unisimon.edu.co (E.N.-Q.); 4 Facultad de Ciencias de la Salud, Universidad Simón Bolívar, Barranquilla 080002, Colombia; henry.gonzalez@unisimon.edu.co; 5 Facultad de Ciencias Básicas y Biomédicas, Barranquilla 080002, Colombia; 6 Facultad de Ciencias Jurídicas y Sociales, Universidad Simón Bolívar, Cúcuta 540001, Colombia; diego.rivera@unisimon.edu.co

**Keywords:** cancer, immunotherapy, solid tumors

## Abstract

Chimeric antigen receptor T cell (CAR T cell) therapy has emerged as a prominent adoptive cell therapy and a therapeutic approach of great interest in the fight against cancer. This approach has shown notorious efficacy in refractory hematological neoplasm, which has bolstered its exploration in the field of solid cancers. However, successfully managing solid tumors presents considerable intrinsic challenges, which include the necessity of guiding the modified cells toward the tumoral region, assuring their penetration and survival in adverse microenvironments, and addressing the complexity of identifying the specific antigens for each type of cancer. This review focuses on outlining the challenges faced by CAR T cell therapy when used in the treatment of solid tumors, as well as presenting optimizations and emergent approaches directed at improving its efficacy in this particular context. From precise localization to the modulation of the tumoral microenvironment and the adaptation of antigen recognition strategies, diverse pathways will be examined to overcome the current limitations and buttress the therapeutic potential of CAR T cells in the fight against solid tumors.

## 1. Introduction

For decades, conventional therapeutic approaches, such as chemotherapy, radiotherapy, and surgery, have been implemented as fundamental pillars in the treatment of various cancer typologies. Most of these interventions have generalized and highly aggressive effects on cells that are in a constant dividing process [1]. Despite the efficacy shown by these modalities, significant challenges persist, including disease recurrence and the rise of intrinsic drug resistance, not to mention potential side effects [2]; therefore, a critical need to explore innovative alternatives that could provide secure and effective solutions in the oncology field has become apparent. This imperative matter has led to the convergence of oncology and immunology, paving the way for the emergence of the field known as immuno-oncology [3]. Contemporary advances in this discipline have revealed novel approaches to the conception of anti-neoplastic therapies based on the enhancement of the innate immune response of the organism. These emergent strategies not only have the potential to improve the life quality of patients but also broaden their survival perspectives [4].

Given the considerable impact of lymphocytes on tumoral progression dynamics, innovative therapeutic approaches have given them greater prominence. These strategies pursue the reactivation and reinforcement of the antitumoral capabilities of lymphocytes through genetic manipulation, giving rise to the known chimeric antigen receptor T cells (CAR T cells). These cells have been genetically modified to express synthetic chimeric receptors capable of recognizing malignant cells, with the purpose of triggering a process directed at the complete eradication of the tumor [5,6].

Over half a century ago, the concept of antitumoral cell therapy was coined by Avrion Mitchison through experimental research in tumoral transplants on murine models. These studies showed that, by transplanting lymph nodes, there was an increase in their size, and they manifested a potentiated immune response. In the past two decades, T cell immunotherapy went from being considered a marginal interest to being recognized as a potential alternative therapy. From the seminal research on the genetic redirection of cytotoxic T lymphocytes to neoplastic cells by Gross et. al. [7], the promise of this approach in the fight against cancer has been regarded with increasing attention, which led to the development of first-generation CARs. Ever since this primary design, the configuration of these receptors has advanced until reaching a fourth generation. Nonetheless, there are still opportunities for refining and optimizing these components, opening the door to a new era of more efficient and safer oncological treatments [8].

In the contemporary context, CAR T cell therapies have experienced significant advancements. Clinical trials using these biomedical technologies have received regulatory approval in countries such as the United States and China, with institutions such as the Food and Drug Administration (FDA) licensing specific drugs for the treatment of acute lymphoblastic leukemia (ALL) and diffuse large B cell lymphoma (DLBCL), among other hematological neoplasms and refractory cancers [9,10]. These successful initiatives have catalyzed a robust expansion in subsequent research and generated significant enthusiasm in this scientific field [11].

However, the applicability of CAR T cell therapies is considerably limited in the case of solid tumors. Numerous studies on a broad range of neoplasms have elucidated intrinsic obstacles that restrict the efficacy of CAR T cells in solid malignancies. Among these obstacles are the identification of specific tumoral antigens that enable the selective recognition of target cells [5], the inherent difficulty regarding spatial localization of neoplastic regions [12], as well as challenges related to the penetration and survival of these cells in hostile tumoral microenvironments [13]. These limitations have been contributing factors to the insufficient performance of immunomodulatory therapies in several clinical trials focused on solid tumors.

In the current research situation, a preeminent focus of interest lies in the development of innovative strategies that can mitigate these complex challenges. The ultimate goal is to achieve an adaptation of CAR T cells that allows them to act effectively and safely in a wide range of malignancies, including solid tumors [10].

The objectives of this review are to rigorously examine the specific challenges that CAR T cell therapy faces in the field of solid tumors and outline emerging therapeutic strategies that could be implemented to increase the clinical efficacy of these treatment modalities in comparison to their performance against hematological neoplasms.

## 2. CART T Cells: Origins and Structure

**Figure 1:**
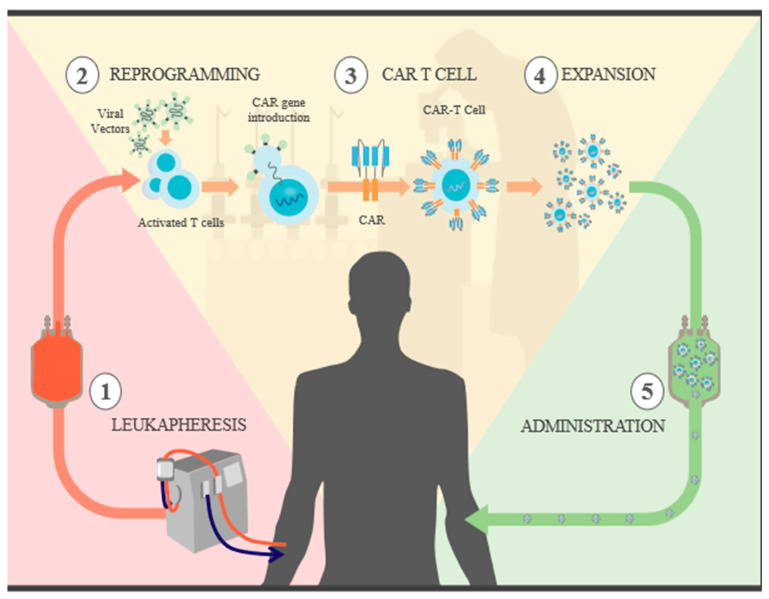
CAR T cell synthesis: 1. recognize specific antigens expressed on tumor cells. Leukapheresis: blood is extracted from the patient, and both CD4+ and CD8+ T cells are selected. 2. Reprogramming: the isolated T cells are cultured and then exposed to antibody-coated beads to activate them. CAR genes are introduced through different viral vectors, which results in permanent genome modification and CAR expression. 3. CAR T cells: now, the T cells are capable of recognizing and attacking specific tumor antigens. 4 Expansion: with the goal of generating a sufficient number of CAR T cells for therapy, the cells go through several rounds of divisions in different culture media. They are then cleansed and concentrated, and a sample is taken for quality control. 5. Administration: prior to the reintroduction of CAR T cells, the patient undergoes “preconditioning” chemotherapy for lymphodepletion, which reduces immunosuppressive cells that could slow the expansion of CAR T cells; in addition to releasing proinflammatory cytokines that promote the activation of CAR T cells, once infused, they are able to initiate their destruction.

CAR T cell therapy represents a pioneering autologous and polyclonal treatment conceived by Rosenberg et al. in 1988 for the management of advanced neoplasms. In this approach, T lymphocytes are genetically modified to express a chimeric antigen receptor (CAR) based on the antibody structure [14]. In this regard, these modified cells are more adept at identifying and eradicating neoplastic cells than their conventional counterparts [15] (see Figure 1).

From a molecular perspective, the basic structure of these CARs amalgamates intrinsic elements such as T cell receptors and antibodies [3]. Specifically, CARs are composed of an extracellular domain responsible for antigen binding [16] and intracellular domains involved in signaling pathways [17]. The extracellular segment, composed of a single-chain variable fragment (scFV), enables T cells to recognize and bind specific tumoral antigens [18]. The subsequent lymphocyte activation is executed by means of intracellular domains derived from the tyrosine-based activation motifs of the immunoreceptor CD3 ζ ITAM [19], thereby facilitating increased affinity and avoiding the restrictions of MHC, which results in optimized tumoral recognition [20].

To complete the CAR architecture, a transmembrane domain and a spacer region, generally derived from immunoglobulins or CD8α, consolidate the connection between the scFV and the intracellular domains [21].

The evolution of CAR designs in clinical trials is categorized into generations. First-generation CARs [22] incorporate antibody-based extracellular signaling domains and an intracellular CD3 ζ signaling domain [17]. However, these receptors lack co-stimulatory elements, limiting their persistence and antitumoral efficacy [23]. Complete cell activation requires at least two different signals: direct TCR signaling and a co-stimulatory pathway, generally mediated by interactions with molecules such as CD28 or CD86, present in antigen-presenting cells (APCs). This co-stimulation can catalyze significant cell responses, including lymphocyte activation and persistence [24,25].

The need for co-stimulation led to the development of second-generation CARs, which incorporated molecules such as CD28 [26] and 4-1BB [27]. These domains potentiate lymphocyte persistence and rapid activation and are essential for robust signaling, lymphocyte proliferation, and epigenetic and metabolic changes in T cells [21]. Despite their achievements, second-generation CARs faced challenges related to their efficacy and safety in certain cancers, which motivated the development of third-generation CARs. This generation incorporates multiple signaling and co-stimulatory domains that augment the effects [28]. Finally, fourth-generation CARs, known as TRUCKS, emerged [8], which included two separate transgenes and expressed CARs from previous generations simultaneously with activating cytokines (such as IL-12) [29,30]. This generation of CARs was introduced to actively modulate the tumor microenvironment and potentiate CAR T cells [31].

## 3. Mechanisms of Action

The activation of CAR T cells is based on a series of molecular and cellular interactions whose specificity is determined by the particular structure of the chimeric receptors. Although the full range of mechanisms involved has not been comprehensively described [32,33], the mode of action of FDA-approved drugs has been established, such as Yescarta and Kymriah [34,35]. These therapeutic procedures begin with the acquisition and later reprogramming of the patient’s own T cells [36], which are genetically modified by means of transgenes that encode CARs specific for CD19 antigens, an exclusive marker expressed on the cell membrane of B lymphocytes [37,38].

The architecture of these CARs comprises a scFV fragment capable of recognizing CD19, which is fused to differentiated intracellular domains, either 4-1BB (in the case of Kymrriah) or CD28 (in Yescarta), and a CD3ζ domain [39]. Once the CAR recognizes and binds to CD19+ cells [40], a series of signaling events are triggered, starting with the phosphorylation of ITAMs and culminating in the activation, expansion, and persistence of modified lymphocytes. Additionally, these lymphocytes acquire effector functions, secrete proinflammatory cytokines and chemokines, and release cytotoxic granules that exert a direct lytic effect over target cells [41].

**Figure 2:**
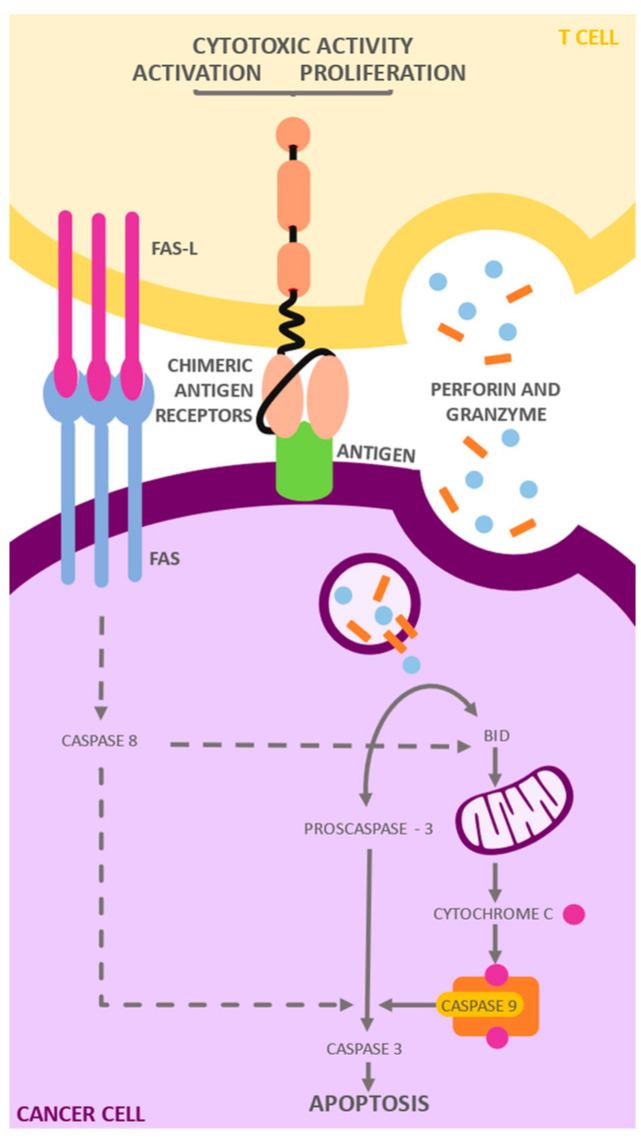
Mechanism of action of CAR T cells: CARs target the CD19 antigen, a molecule expressed on the surface of B lymphocytes. After antigen recognition, CARs transmit cascading signals that promote activation, expansion, persistence, and acquisition of effector functions. These cells then secrete cytokines and inflammatory chemokines that mediate the elimination of target cells through (1) the secretion of perforin and granzymes, and (2) the Fas/Fas-ligand signaling pathway. In the presence of Ca, perforins are activated and are able to be incorporated into the cell membrane of the target cell. Consequently, the perforins and granzymes are internalized through endocytosis. Once inside the vesicle, the perforins form pores that allow the granzymes entry into the cytosol, where they initiate apoptosis. On the other hand, TNF binding to Fas results in the formation of a proapoptotic signaling complex that causes the activation of a cell death effector protease known as caspase 8, which in turn cleaves and activates a caspase cascade that ultimately leads to cell death.

The process of elimination of tumor cells is orchestrated by two main pathways. CD4+ T lymphocytes predominantly release granules that contain perforins and granzymes [42]. In parallel, the activation of the death receptor through the Fas/Fas-ligand signaling pathway (Fas-L) has been postulated as another operative mechanism. Furthermore, CD8+ T lymphocytes exhibit the ability to destroy tumor cells through both pathways, which shows the complexity and redundancy of immune recognition and elimination systems (see Figure 2). In this context, the dynamics and coordination of these pathways reflect the intricate and multifactorial nature of the immune response against tumoral cells [43,44,45] (see Figure 2).

## 4. Causes of Ineffectiveness in Solid Tumors

In the context of solid tumors, there are numerous physiological and biochemical barriers that complicate the efficacy of CAR T cell therapies [46]. Unlike liquid malignancies (see Table 1), multifactorial elements that modulate the therapeutic success are the paucity of ideal tumoral antigens for the therapy [46], the presence of a tumoral microenvironment that exerts an immunosuppressive function [47], and the deficiency of T lymphocyte homing to the tumor site [48]. These obstacles, which act synergistically, contribute to the complexity of the therapeutical landscape of these types of neoplasms. It is imperative to consider that these factors do not work in isolation but are interrelated in a network of interactions that potentially affect the therapeutic outcome. For example, the absence of adequate tumor antigens could be intrinsically related to adaptative changes in the tumor environment, which could influence the efficacy of T lymphocyte homing. In this regard, overcoming these obstacles is not simply a matter of addressing each factor individually but of understanding and modulating the system as a whole to optimize the immunotherapeutic response. Therefore, identifying and overcoming these obstacles would not only catalyze significant advances in the development of more effective immunotherapies but could also represent a paradigm shift in the overall strategies for cancer treatment [49].

### 4.1. Antigens

Cancer cells express a series of molecules, generally of protein nature, called antigens, capable of triggering immune responses. Immune cells, e.g., lymphocytes, have receptor molecules on their surface that bind to these antigens, thereby allowing the start of a sequence of reactions culminating in the destruction of the target [61]; however, due to their mutating behavior, malignant cells often become capable of evading the immune system, which leads to better neoplasm dissemination [62]. For this reason, CAR T cell receptors possess specialized domains that act independently from MHC molecules for the recognition of specific tumor antigens, therefore allowing them to destroy the cells where the antigen molecules are present [63].

**Figure 3:**
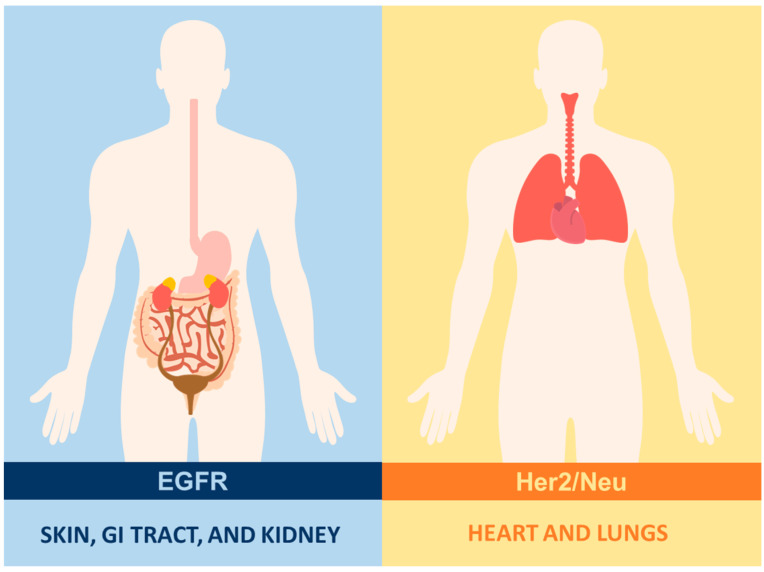
Antigen expression in healthy tissues: in the case of solid tumors, the selection of surface antigens that are exclusive to transformed cells represents a challenge due to their paucity in epithelial cells. Furthermore, these antigens tend to be heterogeneous in distribution and intensity. Such is the case with Her2/Neu, which is expressed in the lungs and the heart, and EGFR, which is found in the skin, digestive tract, kidneys, among others.

Consequently, adequate antigen selection is a key task that makes the difference between success and failure when designing new CARs. In this regard, an antigen frequently used in therapies for liquid neoplasms, such as ALL and DLBCL, is the CD19 molecule [9]. It is expressed in the vast majority of B lymphocytes, including both normal and transformed cells. This feature has led to high levels of effectiveness and generally satisfactory outcomes. However, its lack of specificity restricts its therapeutic potential since it entails serious, and often fatal, adverse effects [64,65]. This was particularly portrayed in 2010 after the death of a patient who was treated with CD19-targeting CAR T cell therapy, although it is worth mentioning that the cause of death is presumed to have been an underlying infection caused by the preconditioning regimens rather than the antigen itself [64,65,66,67] (see Figure 3).

In addition to CD19, other antigens frequently used in liquid neoplasms are CD22 [68] and CD30. The former is expressed on the surface of B lymphocytes, whose use has been considered for the treatment of B-ALL given the multiple cases of antigenic losses occurring during CD19 therapies. On the other hand, CD30 [69] is a member of the tumor necrosis factor (TNF) family [64] and has recently been used as a possible target for CARs thanks to its presence in Reed–Stemberg cells. New therapeutic trials indicate the possible effectiveness of these therapies as well as the probability of reduced side effects due to antigen specificity [69].

However, antigen selection has proven challenging in the case of solid tumors due to the presence of several obstacles. TCR affinity is lower for intrinsic MHC peptides such as tumor antigens compared to pathogen-derived peptides. Therefore, in most cases, it is difficult to isolate T cells whose sensitivity is higher when identifying TAAs (tumor-associated antigens) in comparison to pathogen-derived antigens [70].

Additionally, finding surface antigens that are exclusively expressed in transformed cells is more challenging in the case of solid tumors since, unlike liquid tumors, these are mainly composed of epithelial cell lines, which possess scant surface antigens and increased heterogeneity [71]. Consequently, antigenic loss or tumor escape are possible scenarios [72]. In this regard, although antigens used in solid tumors have managed to transcend from experiment to clinical trial, to date, they have failed to demonstrate efficacy comparable to targets used in liquid neoplasms [18].

This has been evidenced in multiple clinical trials carried out on different types of solid malignancies. For example, in one case report performed in 2010, the transfusion of CAR T cells targeting the Her2/Neu antigen culminated in the death of the patient, which resulted from an assault on normal lung and heart tissues. This event demonstrates how pertinent proper antigen choice is for the safety and success of such therapies [59].

Another relevant case is the EGFR molecule, a tyrosine kinase transmembrane receptor classified as a tissue antigen expressed in the skin, digestive tract, kidney, and other healthy tissues. It is produced in aberrant amounts in certain malignancies such as lung, pancreatic, breast, and colorectal cancer as well as head and neck squamous cell carcinoma (HNSCC) [73,74]. Its presence is associated with decreased survival due to its role in cell multiplication, division, and metastasis. This antigen was used in a phase I clinical trial, where, out of eleven evaluable patients with non-small cell lung cancer (NSCLC), two patients demonstrated partial response and five had stable disease for two to eight months. In addition, permanence of CARs in tissue and blood was observed, accompanied in some cases by minor side effects such as nausea, vomiting, dyspnea, and hypotension [75,76].

In the case of ovarian and breast cancer, overexpression of the transmembrane glycoprotein HER2 has been associated with malignant transformation as well as carcinogenesis and decreased survival. It can also be found in osteosarcomas, GBM, and medulloblastomas [77,78]. Currently, multiple clinical trials using this target are under development, yet drugs such as lapatinib or trastuzumab have generally yielded unfavorable outcomes [79].

The antigen CAIX, which is overexpressed in renal carcinomas [57,80] and absent in normal cells, is another target that has been used in phase I and phase II clinical trials [81]. In a study performed on twelve patients with metastatic renal cell cancer, negative responses to the treatment were observed sometime after the infusion of ten daily doses. These adverse effects were caused by the formation of specific antibodies against the CARs as well as the existence of severe cytotoxicity (grades 2–4) and hepatic infiltration, all of which prevented the completion of treatment in the case of certain patients [82].

Alternatively, tumor antigens expressed during fetal development or at immune-privileged sites [83], e.g., CEA (carcinoembryonic antigen), are present in certain cancers, such as colorectal carcinoma, pancreatic adenocarcinoma, and breast cancer [84], and have been considered for their use in immunotherapies. However, these antigens are often found within the cytoplasm and, therefore, are inaccessible to CARs. Nevertheless, the synthesis of CARs with scFV domains capable of recognizing cytoplasmic antigens circumvents this problem [9].

Finally, antigens expressed in specific tissues or cell lines constitute the most widely used antigen varieties. Although indispensable organs could also be affected, toxicity control is feasible, and, therefore, therapies implementing these antigens do not lead to fatal outcomes in most cases [85]. For example, in a clinical trial using PSMA (prostate-specific membrane antigen), an antigen expressed on malignant prostate cells, greater antitumor efficacy and effective elimination were observed [59] (see Table 2).

### 4.2. Tumor Microenvironment

The tumor microenvironment represents a complex and heterogeneous intratumoral structure that houses a series of both neoplastic and non-neoplastic cells. Within this cellular compendium, fibroblasts, adipocytes, pericytes, immune cells, and stromal cells reside [86]. In addition to these cells, the tumor microenvironment also comprises blood vessels, extracellular matrix, and signaling molecules that perform fundamental roles in tumor pathogenesis [87].

The dynamic interaction between tumor cells and the tumor microenvironment has a determining influence on the malignant phenotypic expression of neoplastic cells. These reciprocal interactions facilitate the acquisition of characteristics critical for tumor progression, showing that the tumor is not simply a passive spectator but an active and essential agent in the promotion of carcinogenesis and the development of advanced neoplasms. Likewise, the suppressive nature of the tumor microenvironment represents a significant challenge to the efficacy of CAR T cells targeting solid tumors [88].

Within this complicated matrix, the cells that migrate to the tumor environment face numerous barriers. These include cells and molecules with immunosuppressive properties [89], functional inhibitions derived from the interaction between CAR T cells and tumor cells [90], physical barriers presented by the tumoral stroma, and a physiologically challenging environment marked by hypoxia and elevated levels of toxic metabolites, such as nitric oxide [91,92].

#### 4.2.1. Immune Checkpoint Inhibitors

The TME is an intricately complex and dynamic system in which cancer cells and immune components interact in constant co-evolution. Within this complex landscape, tumor cells have the capacity to generate ligands that inhibit immune checkpoints in response to the presence of antitumorigenic T cells, in particular cytotoxic T lymphocytes (CTL) [93]. This adaptive response is a manifestation of the complexity and plasticity of the TME.

After infiltrating the TME, T lymphocytes start secreting cytokines, such as interferon-gamma (IFN-γ), which trigger the expression of molecules such as PD-1, CEA CAM 1, and CTLA-4 [94]. This molecular cascade, consequently, facilitates the evasion of immunological responses. These inhibitory molecules interact with checkpoint receptors on T cells, such as TIM3, LAG3, CD160, and VISTA, leading to cellular dysfunction by means of several mechanisms [95,96]. For example, LAG3 negatively modulates class II MHC molecules, while TIM3 induces apoptosis and inhibits T cells after it binds to specific immunoglobulins such as galectin-9 [97,98].

From a complexity perspective, it is essential to consider that CAR T cells, designed to combat tumors, interact with this sophisticated immunosuppressive TME in ways that have not been fully elucidated yet [92]. Theories suggesting that CAR T cells may immediately lose their cytolytic and secretory functions after entering the TME have been postulated. This is called a “hypofunctionality” state. However, we still face the challenge of determining the specific factors that trigger these effects [99]. It is imperative to understand these mechanisms from a holistic and multidisciplinary approach to unravel the true nature of the interactions with the TME and develop more effective therapies.

#### 4.2.2. Physical Barriers

The architecture of the tumor stroma represents a complex physical barrier that may hinder the effective penetration of CAR T cells. This stroma is composed of an extracellular matrix (ECM), blood and lymphatic vessels, immune cells, mesenchymal cells, and fibroblasts, which configure a dense meshwork that provides difficult access to effector cells of the immune system [100]. In certain cases, the compaction of this structure can be such that it makes the tumor practically impervious to interventions with CAR T cells, which is especially problematic given that a greater presence of these cells usually correlates with a more favorable prognosis [101].

The complexity of this scenario is accentuated due to the biochemical and mechanical interactions between stromal and cancer cells. These interactions precipitate a series of metabolic, genetic, and morphological changes, transforming cells into pathological forms that favor tumor progression and metastasis. Specific stromal components, such as the ECM, are rich in modulating elements, such as proteoglycans or glycopeptides, that influence the remodeling of immune responses during the carcinogenesis process [102].

In this context, fibroblasts experience a phenotypical transition to cancer-associated fibroblasts (CAF). In this altered state, CAF expresses effector molecules such as fibroblast activating protein (FAP) and stromal-derived factor 1a (SDF1A). These molecules, in synergy with CAF, can induce ECM degradation and collagen cross-bridging, thus facilitating tumor progression [89].

It is postulated that integrin overexpression in CAFs could trigger a series of protumor events, e.g., potentiation of oncogenic growth factor receptor (GFR) signaling, colonization of metastatic sites, and the facilitation of the survival of tumor cells adjacent to the extracellular matrix [103]. Additionally, alterations in the stroma can contribute to the processes of carcinogenesis and metastasis by coordinating angiogenesis [104].

This panorama illustrates the intricate web of cellular and molecular interaction that underlies the biology of tumor stroma, which adds an additional layer of complexity to the optimization of CAR T cell therapies. In this context, a deeper understanding of tumor ecology and its underlying mechanisms is imperative for the development of more effective and precise therapies.

#### 4.2.3. Metabolic Conditions

The TME is a dynamic and complex system composed of neoplastic, stromal, and immune cells, as well as a variety of soluble molecules. Its multifaceted nature and reciprocated interaction between its components perform fundamental roles in tumor progression and resistance to therapies, including immunotherapies [105].

In particular, a metabolic condition that separates tumor cells from normal cells is their preference for aerobic glycolysis to obtain energy even in the presence of oxygen, a phenomenon known as the Warburg effect [106]. It is essential to recognize that not only tumor cells adopt this metabolic mechanism. Effector T lymphocytes, which are crucial for antitumor immune responses, also depend on glucose to fuel their expansion [107]. This metabolic coincidence leads to competition for nutrients in tumor regions with nutritional deficiencies. As a result, metabolic competition can compromise the function and proliferation of T cells, favoring tumor progression and metastasis.

Lactic acid, a byproduct of glycolysis, accumulates in the TME, which lowers the local pH levels [108]. This metabolic acidosis is not the only pathway that has toxic properties for T cells and cytokines; metabolites derived from other metabolic pathways, e.g., glutaminolysis, also generate immunosuppressing effects that can compromise CAR function [109].

Additionally, a pronounced feature of the TME is its hypoxia resulting from deficient angiogenesis and the insufficiency of progenitor cells to generate adequate vasculature [110]. Hypoxia has tumorigenesis-promoting effects by inducing glycolysis and supporting neoplasmic growth. While the impact of hypoxia on immune cells still needs to be explored in depth, it has been identified that hypoxia can stabilize HIF-1α in TAMs, which leads to an increase in the secretion of protumor molecules and enzymes that degrade the extracellular matrix, thus facilitating tumor invasion [111].

The competition for amino acids in the TME, especially L-arginine and tryptophan, also emerges as a significant challenge for the persistence and function of lymphocytes. These amino acids are essential for numerous cellular and metabolic processes, and their scarcity, exacerbated by the enzymatic overexpression of malignant cells (such as IDO and TDO), can cause immunological dysfunctions, compromising the ability of T cells to recognize and attack tumors [112,113].

### 4.3. Trafficking into the Tumoral Area

One of the difficulties CAR T cells face in solid tumors is proper trafficking to the neoplastic area [114]. Unlike liquid tumors, where therapeutic targets are easily accessible because they are either circulating in the blood or lymph or found in the bone marrow, solid tumors are found in tissues; therefore, CAR T cells must be trafficked to these specific sites. Unfortunately, this transport can be affected by several factors [115].

On the one hand, CAR T cells and transformed cells in liquid tumors share a common hematopoietic origin, which explains why these cells tend to migrate to similar areas. On the other hand, solid tumors have barriers that limit the migration of CAR T cells to their respective targets independently of the amount administered [116].

Although it has been possible to observe the successful migration of CAR T cells to the neoplastic stroma and their subsequent accumulation in that area, the tumor parenchyma still remains largely inaccessible. The inability to infiltrate this region has been attributed to a series of vascular phenomena, such as the existence of migration regulatory agents, e.g., the TME3 cellular endothelium, which is able to control the cytokines synthesized by the TME in addition to functioning as a selective barrier that prevents infiltration [117]. Under normal conditions, the endothelium is responsible for secreting substances such as ICAM1 and VCAM1 [118] to promote the migration and extravasation of inflammatory cells; however, the carcinogenic endothelium inhibits these molecules in order to evade immune responses. It also stimulates the release of escape promoters such as ALCAM (activated leukocyte cell adhesion molecule), replaces chemokine receptors, and induces integrin-dependent arrest in T cells. On the other hand, vascular elements such as HEV (high endothelial venules) have been linked to the effective migration of immune cells to solid tumors due to their optimal adaptation for the recruitment of T cells in certain malignancies such as melanomas and breast cancer [119,120].

During lymphocyte migration, adhesion molecules such as selectins, integrin, and chemokine receptors influence cellular trafficking. The binding of these adhesion molecules to cytokines produces the phenomenon of lymphocyte rolling followed by a cascade of biochemical events that finally leads to the penetration of the endothelium and trans-endothelial migration to the tumor area [121].

Likewise, the secretion of substances such as chemokines is imperative for immune cell migration. These molecules, which are overexpressed in stromal, cancerous, and immune cells, play fundamental roles in tumor growth and remodeling of the TME through the production of chemotactic gradients, which ultimately regulate cellular trafficking [122].

However, in tumor and stromal cells, cytokine secretion is decreased, thus increasing the chance of evading immune responses [116]. Furthermore, these cytokines change in such a way that the receptors expressed on T lymphocytes cannot recognize them [123]. In addition, transformed cells in pancreatic, ovarian, and breast cancers have the ability to secrete cytokines, e.g., CXCL12, that inhibit CAR T cell proliferation and migration, thereby interfering with cell delivery toward the tumor zone [124,125].

In addition to the inhibitory properties of solid tumors, manufacturing defects may influence CAR T cell homing. Newly isolated CAR T cells usually have numerous receptors targeting different chemokines, which can be altered by prolonged ex vivo amplification. On the other hand, the loss of certain components, such as the enzyme heparanase, which degrades heparan sulfate located in the tumor stroma, during CAR programming can increase the ineffectiveness of lymphocytes when penetrating into the parenchyma [126].

**Figure 4:**
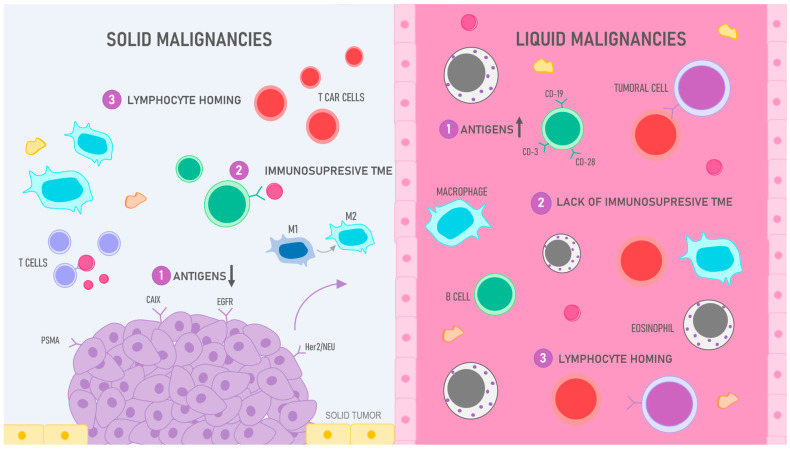
CAR T cell efficiency: liquid vs. solid malignancies: 1. Antigens: while the target antigens in liquid malignancies such as leukemia and lymphoma are efficient, solid tumors pose a challenge due to their scarce and heterogenous expression of antigens; various antigens like CAIX, EGFR, Her2/neu, and PMSA, among others, have been tested; however, the results have not been completely satisfactory, particularly in contrast with CD-19 antigen, used to treat B cell malignancies, the arrows pointing down in solid tumors represent the scarcity of antigens in these malignancies and, in contrast, the arrow pointing up in liquid neoplasia reflects the availability of these surface molecules in these cancers. 2. Immunosuppressive TME: the hostile tumoral microenvironment (TME) in solid tumors has hampered the development of successful CAR T cell therapy since this environment not only promotes tumoral development but also has the ability to deactivate immune cells, therefore obstructing the labor of CAR T cells; in contrast, liquid neoplasia does not present these issues as the cells are “fluid” and circulate within blood or lymphatic vessels. 3. Lymphocyte homing: finding the target antigen in leukemias and lymphomas is not a particularly challenging task for T cells since these cells can be found circulating in the bloodstream; however, in solid tumors, CAR T cells must be trafficked to specific sites, penetrate into the tumoral stroma, and evade multiple suppressing molecules to reach their target.

Lastly, it is worth highlighting, among the limitations of CAR T cell therapies, that, despite all the interest in this therapeutic approach, its application is very confined. Most clinical trials are performed in the USA (*n* = 377), China (*n* = 636), and Europe (*n* = 58). In 2021, only approximately 2500 patients received CAR T cells; according to the EBMT registry, these numbers seem particularly low when compared to the proportion of oncologic patients with indications for CAR T cell infusion, leading to the conclusion that about 99% of the potential candidates for the treatment did not receive it [127]. The answer to this specific issue resides in the high cost of the therapy, estimated to be USD 500,000, and the intricate and long process of manufacturing that usually takes 21–35 days. If the goal is to establish CAR T cells as a future standard treatment for cancer, it is necessary to develop more affordable and practical approaches for the public (see Figure 4).

## 5. Optimizations to Increase CAR T Cells’ Effectiveness in Solid Tumors

### 5.1. Specificity and Toxicity

The toxicities caused by the lack of specificity of the targets used in solid neoplasms so far, as well as the possible relapse of the disease caused by tumor heterogeneity, could be avoided thanks to various approaches in development. The most desirable solution to resolve on-target off-tumor toxicities may be finding antigens expressed exclusively in cancer cells [71,128]. Fetal antigens such as fetal acetylcholine receptors have been proposed for rhabdomyosarcomas treatment. Likewise, glycosylated MUC 1 has been proposed for adenocarcinomas. In addition, screening for appropriate antigens before cell infusion could be an excellent tool against neoplasm heterogeneity [129].

Currently, several techniques focused on increasing specificity and reducing tumor escape are being implemented on CAR T cells. Different models consisting of chimeric receptors with multiple targets have been proposed, of which dual CARs [130], TanCARs [131], and iCARs [132] are the principal biospecific CARs. Dual CARs express two receptors, each targeting a specific antigen [133]. In this context, simultaneous expression of both antigens is required for proper T cell activation, thereby increasing therapeutic specificity [134]. On the other hand, TanCARs, or tandem CARs, possess a receptor capable of recognizing different antigens due to contiguous antigenic recognition domains [135]. This model allows the synergistic activation of extracellular domains when both antigens are bound. Even though this model is less specific than dual CARs, it can persist even after one antigen is lost, thus preventing tumor escape [136]. Finally, iCARs, or inhibitory CARs, are co-administered with conventional CARs but recognize different antigens. When the target cell expresses two determined tumor antigens, the iCARs bound to PD1 or CTLA-4 cytoplasmic domain are activated and transmit negative signals that decrease CAR T cell function with the goal of reducing the elimination of proteins that are expressed in normal tissues but reduced in tumors [137].

Similarly, modulating receptors have been designed with SynNotch technology. These receptors are composed of one extracellular antigenic recognition domain and one intracellular “Notch Cleavage” domain [138] fused with a transcription factor [138]. Once the T cell binds to a first antigen, the SynNotch system induces the expression of a secondary CAR that will modulate cellular activity in the presence of a second antigen [139].

Another approach to increase therapeutic specificity is focused on natural killer cells (NK cells) [140], which express receptors capable of distinguishing normal cells from transformed cells [141]. In this regard, NKG2-D recognition domains and their ligands, numerous heat shock proteins overexpressed in neoplasms, have been used in numerous types of tumors [142,143]; once the union is created, this produces the activation of the T cell and, with it, an increase in antitumor activity [144]. On the other hand, alternatives such as CAR switches based on antibodies expand the possibility of having multiple targets in one construct by incorporating a “tag” in a specific anti-TAA antibody [145]. Subsequently, these are redirected to eliminate those cells that express the antigen in question. However, it has been shown that the effectiveness of this procedure depends in turn on factors such as the dose of cells administered and the ability of CAR T cells to penetrate the tumor niche.

Lastly, there are modifications destined to control T cell overactivation. The implementation of “suicide genes” has gained prominence in recent years [146]. This strategy allows the selective elimination of CAR T cells that were genetically engineered to code for genes that transform non-toxic molecules into toxic ones, which can be used to induce apoptosis [147]. Likewise, drug-induced elimination systems have also been designed. One such approach is the rituximab-induced elimination system, wherein engrafted T cells are turned into antibody ligands after the administration of rituximab [148].

### 5.2. Tumor Microenvironment

As previously stated, the tumor microenvironment represents an obstacle to CAR T cell therapies. After infiltration, T cells suffer a functional switch that inhibits their antitumoral activities, thus hindering tumor suppression [149]. The addition of co-stimulating molecules such as CD28 [150] or 4-1BB [26] has been proposed to circumvent this problem. These molecules extend T cell activation, prolonging its persistence. In addition, the concept of “switch receptors” has been proposed. These receptors are capable of converting inhibitory signals into stimulators. Furthermore, constructs of CARs with co-stimulatory domains capable of activating specific pathways that lead to IL-Rrb release after antigen recognition have also been conceived [151].

Other models called remote controlled CARs are only activated in the presence of adaptor molecules designed to become specific targets that can be modified after therapy administration, a property that is especially useful for universal CARs [152]. In addition, this modality could be developed faster than customized varieties of these receptors [153,154]. Similarly, recent clinical trials have yielded promising results regarding the efficacy of GD2 CARs after transcription factor JUN addition, obtaining improvements in T cell resistance and antitumor efficacy in both liquid and solid neoplasms [154]. Moreover, studies have also delved into the possible external activation of CAR T cells in lymphoid tissues by antigen-presenting cells (APCs) and the combination of these enterotoxins in order to increase the expansion and antitumor responses [155].

One of the most popular therapeutic strategies to combat immunosuppression involves fourth-generation CARs or TRUCKs [24,156,157], which are specially designed to produce and secrete proinflammatory cytokines and other transgenic products, such as IL-15 [158], IL-18 [159], and IL-21 [160]; however, IL-12 is the most prominent immunomodulator. This cytokine facilitates the conversion of suppressor cells and increases T-cell-mediated cytotoxicity. Furthermore, cells that express IL-12 are capable of polarizing Th1 lymphocytes in order to attract other immune cells and promote the elimination of tumor cells that are “invisible” to CAR T cells. Likewise, this method can also be used for the selective delivery and release of other elements with local effects or whose toxicity is too high for systemic administration [161,162]. Several clinical trials have corroborated the success of TRUCKs and their positive effects on persistence, survival, and cytotoxic activity [163].

**Figure 5:**
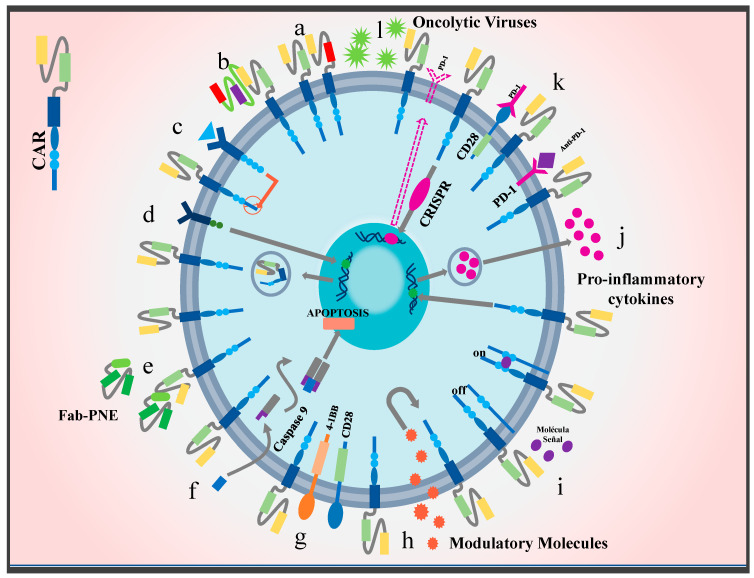
Novel strategies to improve CAR T cell efficacy in solid tumors: currently, a variety of techniques focused on increasing specificity and restricting tumor escape are being implemented. Chimeric receptor models with multiple targets have been proposed, such as (**a**) dual CAR T cells, (**b**) TanCARs, (**c**) iCARs, (**d**) SynNotch CARs, (**e**) switch CARs, and (**f**) CARs with suicide genes. In addition, modifications that improve TME penetration have been introduced. This class includes the co-stimulatory molecules (**g**) CARs 4-1BB and CD28. Likewise, CARs that are activated or inactivated through signaling molecules have been designed, e.g., (**h**) CARs switch receptors and (**i**) remote controlled CARs. To resist the harsh microenvironment, T cells have been modified into (**j**) CARs TRUCKS to produce and secrete proinflammatory cytokines such as IL-15 and IL-18. (**k**) CARs that combine anti-immune therapy checkpoints have also been designed, for example, the addition of CARs combined with anti-PD-1 molecules or the combination of an endodomain with a CD28 molecule and a PD-1 exodomain. Moreover, the use of the CRISPR/Cas9 system has been proposed in order to eliminate genes responsible for the expression of immune checkpoints such as PD-1 or CTLA-4. Finally, to improve delivery and infiltration into the neoplastic area, therapies have been developed that use (**l**) oncolytic viruses for their ability to be deposited in the tumor area.

One of the most notorious effects that tumors have on the immune response is the modulation of immune checkpoints [164]. In this line, the tumor microenvironment is a major player in the alteration of the function of these control points, resulting in anergia, general depression of T cell activity, and, consequently, tumor progression [165]. For this reason, new therapies include antibodies directed specifically toward checkpoint receptors such as PD-1, LAG3, TIM3, VISTA, and CTLA-4 [166,167]. This combination has yielded promising results in solid tumors. For example, in one study on hepatocellular carcinoma, the addition of anti-PD1 and co-stimulatory molecule CD28 resulted in better antitumor efficacy and avoidance of second-generation CAR anergy [90] (See Figure 5). In addition, the CRISPR/CAS9 system has been used to remove genes responsible for immune checkpoints, such as PD-1 and CTLA-4 [168,169]. However, the combination of multiple inhibitor systems is necessary to achieve adequate suppression since the presence of receptors differs according to cell type. Currently, ipilimumab, nivolumab, and pembrozulimab are FDA-approved immune checkpoint blockers [170]; however, these therapeutic techniques continue under development since the mechanism of action of some checkpoints has not been elucidated yet. Also, some patients have developed resistance and autoimmune syndromes, which warrants further optimization [171].

Similarly, the results of multiple clinical trials have shed some light on the association between intestinal microbiota composition and responses to immune checkpoint inhibitors (ICI). This evidence has led to the formulation of certain hypotheses based on the modulation of the microbiota as a tool to modify TME in conjunction with ICI, increase the antitumor efficacy of CAR T cells, and reduce adverse immune effects [172].

In another vein, the cellular metabolism in the TME suffers changes that promote carcinogenesis. On the one hand, the heightened glycolytic and anabolic activities reduce the glucose levels in the TME [173]; therefore, the hypoxia-induced factor 1a (HIF-1a) has been considered as a potential therapeutic target since it stabilizes glucose levels [174]. This transcription factor induces the production of elements such as S-2 hydroxyglutarate, a molecule associated with epigenetic changes and increased expression of IL-2. This cytokine potentiates CD8+ T cell cytotoxicity [175]. On the other hand, the inhibition of IP3 prevents the initiation of the glycolytic pathway through its calcium-ion-blocking effect [176].

Contrary to glucose levels, the MTE is characterized by a high concentration of reactive oxygen species (ROS) that exert suppressive effects on immune cells [177]. For this reason, the enzyme catalase has been included in clinical trials and has shown favorable effects on cellular antitumor functions [178]. Likewise, amino acid depletion in the tumor area is one of the greatest immunosuppressants; therefore, administration of cyclophosphamide or fludarabine has been used to inhibit amino-acid-metabolizing enzymes such as IDO or TDO [179] to restore protein concentrations in the microenvironment [112,180]. Similarly, negative dominant TGF-b receptors have also been implemented due to the association between TGF-b and lymphocyte inhibition, tumor invasion, and metastasis [181]. Finally, since prostaglandin E2 seems to depress lymphocyte activation, it has been considered desirable to disrupt this pathway in order to improve both antitumor function and activation of CAR T cells [182].

### 5.3. Trafficking and Infiltration into the Tumoral Area

Unlike hematological malignancies where the target cells are accessible to CAR recognition [183], the location, abnormal cytokine profile, and physical barriers such as tumor stroma or neovascularization make solid tumors a problematic target in terms of T cell delivery and infiltration [184]. Since lymphocytic cells have no affinity for cytokines expressed in the tumor microenvironment, new receptors targeting TME-specific ligands have been proposed. This principle has been corroborated by studies that used transgenic receptors [185] such as CXCR2, CCR2b, CXCR3, and CCR4. In these studies, CAR T cells were more effectively delivered to different kinds of solid tumors, including melanomas [186], neuroblastomas [187], ovarian cancer [188], renal cancer [189], prostate cancer [190], and mesotheliomas [191]. In this context, CD8+ lymphocyte recruitment is increased, leading to eventual tumor regression in some cases. However, due to tumor heterogeneity, this approach requires extensive background knowledge about the cytokine profile found in each cancer type.

Other therapeutic options consist of modifications in the ligands secreted by the microenvironment [192]. In this respect, low radiation doses and phosphoramide have been used to induce the expression of CXCR4 and CXCL-12 [193], block inhibitory cytokines and receptors such as CXCL12 and CXCL15 [194], and reduce the expression of the endothelin B receptor, a molecule that restrict immune cell infiltration in ovarian cancers [195] Currently, experimental studies in murine models have shown encouraging results on pancreatic cancer through the negative regulation of pro-tumor cytokines. Furthermore, clinical trials with CAR/RIAD T cells (regulatory subunit 1 anchoring disrupter) have found similar outcomes through the inhibition of protein kinase A, a suppressor of lymphocyte receptors [196]. 

Another strategy to augment cell trafficking is the combination of immune therapy with oncolytic viruses (OVs), which are capable of being deposited specifically in the tumor region [197,198,199]. In addition to their specific tropism, OVs can be modified to attract T cells to the TME through cytokine secretion [200]. Current clinical trials using adenoviruses equipped with RANTES (CCL15) and IL-15 have obtained favorable results in causing direct lysis of infected cancer cells and increasing T cell migration, survival, and growth [201]. Furthermore, by implementing these methods, any neoplasm could produce cytokines and growth factors that promote “trafficking” regardless of their origins, thus ruling out the need to study the cytokine profiles of each tumor. However, more clinical trials that use different types of viruses (e.g., adenovirus, herpesvirus, retrovirus, or poxvirus, among others) in both primary and secondary tumors are needed [202].

An alternative to improve trafficking effectiveness is local delivery of CAR T cells [203,204,205]. According to the location of the neoplasm, cell infusions have been performed directly into the hepatic artery in colorectal cancer [206], breast cancer using CAR anti-c Met T cells [207], intracavitary and intraventricular in glioblastomas where it was observed that while the first option managed to reduce tumor diameter, the second managed to dramatically reduce the size of all intracranial tumors [208], without presenting relevant toxicities in any of the registered cases. Other clinical trials have taken these procedures further by using microscopic titanium or “nitinol” plates loaded with CAR T cells, depositing them in the tumor area and resulting in decreased neoplastic development [209].

Once immune cells reach the neoplastic zone, physical barriers such as stroma or microenvironment-induced neovasculature represent challenges for tumor parenchyma infiltration. The addition of ECM-degrading enzymes such as heparanase or relaxin has been proposed [210]; however, during the manufacture of CAR T cells, functional changes that affect heparanase secretion occur, which led to the development of CAR T cells that express HPSE, thus increasing matrix degradation, infiltration, and anti-cancer effects. Similarly, the combination of CAR T cell therapy with junction openers (JOs) has facilitated infiltration thanks to its ability to self-dimerize and expand communicating junctions in union with desmoglein 2.

In addition to the tumor stroma, the TME vasculature may represent a complication for T cell therapy. For this reason, techniques aimed at eliminating or normalizing it have emerged in order to restrict the flow of nutrients to tumor tissue and simultaneously increase immune infiltration [211]. One of the main targets is the molecule VEGF-2 expressed in pro-tumor endothelial cells. Therapy targeting this molecule produced vascular repressions with successful infiltrations and tumor regression in experimental models in which CAR T cells employed IL-12 [212]. Similarly, Angiogenic ligands, e.g., echitastin, which is capable of binding anb3 integrins expressed in tumor epithelium, have been incorporated into CARs. Consequently, Te-CARs have been developed, whose activities, in addition to being specific, have caused tumor regressions in pancreatic, ovarian, and breast cancer [213].

Furthermore, other applications for CAR T cells have been considered outside oncology. Currently, these immunotherapies are being tested in a different range of pathologies with limited therapeutical options or refractory cases, from refractory systemic lupus erythematosus through the induction of B cell aplasia and hypogammaglobulinemia as well as other autoimmune disorders [214], fibrosis of various tissues including the myocardium, mainly through genetic ablation of activated fibroblasts, and removal of senescent cells to improve age-related conditions and infectious diseases such as human immunodeficiency virus (HIV) via targeting CD4+ cells [215]. However, the research is far from over. Currently, the expanding interest has led to considering CAR T cell applications in different fields from cardiology to pneumonology, endocrinology, nephrology, and many others, leading to the notion that perhaps this is only the tip of the iceberg for immunotherapies and specifically CAR T cells.

## 6. Conclusions and Future Perspectives

CAR T cell therapies have emerged as revolutionary advances in oncology and regenerative medicine. These therapies take advantage of the endogenous mechanisms of the immune system to identify and eradicate tumor cells with specificity, potentially offering a curative outcome in certain patients. These properties set them apart from conventional therapeutic interventions that often have a less specific spectrum.

From the perspective of complexity, the rise of CAR T therapies shows a paradigmatic transition in oncoimmunology, reflecting the inherent interconnection between biological systems and the adaptability of modified cells. Despite the noticeable success, particularly in hematological neoplasms, challenges regarding universal use still remain. On the one hand, the need for clinical studies with broader cohorts is indispensable to assess the robustness and replicability of the benefits. Since solid tumors impose specific barriers, a deeper understanding of the tumor microenvironment and immune dynamics within this context is essential.

The manufacturing and scalability of these therapies also represent significant challenges. Therefore, seeking strategies to universalize production could be the key to democratizing access and reducing associated costs.

In spite of the aforementioned challenges, the adaptability and evolution of CAR T cell therapies have shown resilience. The current limitations are not considered to be overwhelming barriers but opportunities for innovation and refinement. The constant optimization of these therapies, either through genetic engineering, novel antigen identification, or structural modification, aims to potentiate their effectiveness and security.

Although CAR T cells were originally focused on neoplasms due to their high incidence and morbimortality, the versatility of this technology is being explored in various other fields. Current research is venturing into their potential use in autoimmune and chronic diseases as well as infections, suggesting a broad and promising horizon for this therapeutic modality in multiple branches of medicine.

## Figures and Tables

**Table 1:** Clinical studies in liquid neoplasms and solid tumors.

Cancer Type	CAR T Model	Sample	Duration	Results	Reference
ALL	*Kymriah*	97 patients	≥3 months	ORR at 82%, CR at 62%, SR after 18 months at 70%, and CRS in 70% of cases, of which 48% were admitted to the ICU.	Fowler et al. [50]
B-ALL	*Tisagenlecleucel*	75 patients	3 months	ORR at 81%, SR at 73% on average over 12 months, CRS in 77% of cases, and neurological events in 40% of patients.	Mueller et al. [51]
ALL	*CTL019*	59 patients	1 session	CR in 93% of patients, SR at 79%, RFS at 76%, and CRS prevalence at 88%.	Maude et al. [52]
ALL	*CTL019*	30 patients	1 month	RR at 87%; CRS in 28–30 of patients; neurotoxicity and B cell aplasia were observed	Maude et al. [53]
LBCL	*Axicabta gene Ciloleucel*	101 patients	1 session	RR at 58%, neurological events in 32% of patients, and CRS grade III in 11% of cases.	Neelapu et al. [54]
NHL	*Lisocabtagene maraleucel*	344 patients	2 sessions	CRS was observed in 73% of individuals, neurotoxicity was observed in 23%, NHL patients obtained 75% ORR and 55% CR, while DLBCL patients obtained an ORR at 80% and CR at 59%.	Abramson et al. [55]
DLBCL	*Tisagenlecleucel*	93 patients	14 months	ORR at 53%, CR in 40% of cases and PR in the remaining 12%, RFS at 65%, CRS in 22% of patients, neurological adverse effects in 12% of cases, cytopenias in 32% of individuals, and prevalence of 20% in terms of infections.	Schuster et al. [41]
DMG	*T CAR Anti-GD2*	5 patients	-	Drastic decrease in tumor size with a reduced number of residual GD2 glial cells and few adverse effects.	Mount et al. [56]
Renal Carcinoma	*T CAR Anti-CAIX*	12 patients	10 sessions	Antigen-specific effects; reduced hepatotoxicity levels at low doses; no clinical effects recorded.	Lamers et al. [57]
Ovarian Cancer	*T CAR Anti-FR*	14 patients	1 session	No tumor reduction was observed in patients, low levels of persistence and high tolerance.	Kershaw et al. [58]
Prostate Cancer	*Anti-PSMA dTc T CAR*	5 patients	2 weeks	2 out of 5 patients achieved RP regardless of dose size administered with IL-2 depletion; no toxicities or reactivity were recorded	Junghans et al. [59]
Sarcoma	*T CAR Anti-HER2*	19 patients	6 weeks	There was persistence for a period of approximately 6 weeks; without significant toxicities, 14 of the patients had stable disease and an OSR of 10.3 months	Kawakama et al. [60]

**Table 2:** Characteristics of antigens used for the development of CAR T cell immunotherapies.

Antigen	Tissue Expression	Cancer Type	Adverse Effects	Clinical Phase
CD19	Physiological and transformed B cells	ALL, DLBCL, NHL, CLL, and other B cell malignancies.	CRS, CRES, B cell aplasia.	Phase I
CD22	B lymphocytes	B-ALL and B cell lymphoma	CRS and neurotoxicity	Phase I
CD30	Transformed B and T lymphocytes (expression is reduced in normal cells)	ACLC, HL, and T cell lymphomas	Significant adverse effects have not been observed	Phase I and phase II
Her2/neu	Widely expressed in physiological cells	Oral, Gastric, lung, and breast cancer	High levels of cytotoxicity	Phase I
EGFR	Skin, GI tract, Kidneys, and other normal tissues	HNSCC and lung, pancreatic, colorectal, and breast cancer	Nausea, vomit, dyspnea, or hypotension	Phase I
HER2	Widely expressed in normal tissues	Ovarian and breast cancer, osteosarcoma, GBM, and medulloblastoma	Lethal cytotoxicity and CRS	Phase I
CAIX	Malignant kidney cells	Renal carcinoma	Severe cytotoxicity and hepatic infiltration	Phase I and phase II
CEA	Colon, stomach, tongue, cervix, and prostate	Colorectal carcinoma, pancreatic adenocarcinoma, and breast cancer	Fever, Leukopenia, and grastritis	Phase I
PSMA	Prostate	Prostate cancer	Well-tolerated; can course with neurotoxicity in some cases	Phase I
